# Things That Become Visible, for a While, Can Leave a Residue: Comment on "Ensuring Global Health Equity in a Post-pandemic Economy"

**DOI:** 10.34172/ijhpm.2022.7756

**Published:** 2023-01-18

**Authors:** Nele Jensen

**Affiliations:** Department of Global Health & Social Medicine, King’s College London, London, UK.

**Keywords:** COVID-19, Pandemic, Equity, Public Health, Social Justice, Capitalism

## Abstract

Professor Labonté’s editorial is an important intervention that reiterates the stark socio-economic and health inequities that were exposed and perpetuated during the coronavirus disease 2019 (COVID-19) pandemic to call on the public health community to hold politicians to account for their promises of ‘building back better.’ The editorial makes present how quickly pandemic promises seem to have become dislodged by an ostensibly endless cycle of political and economic crises. But it also expresses a hope that lessons from the pandemic will eventually serve to challenge prevailing (economic) policy orthodoxy and feed a collective demand for more progressive social, economic and environmental justice-oriented politics.

 Writing in the early months of the coronavirus disease 2019 (COVID-19) pandemic, as images of the mass migration of city-dwelling Indian labourers to their home villages visualised the radically uneven distribution of health, social and economic risks posed by both the pandemic and the containment measures implemented in response,^1^ Indian writer Arundhati Roy compared the pandemic to both, a “chemical experiment that suddenly illuminated hidden things” but also a chance to “rethink the doomsday machine we have built for ourselves.”^2^ Two and a half years later, Professor Ronald Labonté’s editorial^3^ is a clarion call aimed at the public health community to mobilise the lessons learned from the COVID-19 pandemic as evidence to ensure that empty post-pandemic promises of ‘building back better’ are superseded by a collective effort to foster a ‘sustainable caring economy.’ But it is also a timely reminder of how quickly such post-pandemic promises have become overshadowed by a seemingly endless cycle of political events and snowballing economic crises. As the United Nations warns of rebounding COVID-19 infection rates coalescing with an intensifying climate emergency, rising inflation, a severe energy crisis and spiralling food insecurity into ‘cascading and intersecting global crises threatening human survival,’^4^ Professor Labonté’s editorial highlights the need to be aware of the politics of attention and neglect that legitimise a perpetual cycle of stopgap solutions in the name of crisis management at the expense of more radical structural change.^5,6^

 Labonté’s baseline argument is that the pandemic has exposed how health inequities are grounded in socio-economic inequities that, in turn, result at least partly from economic policies. I should note here that I am not an economist – I am a medical doctor and social scientist whose work draws on pragmatist philosophy, postcolonial science studies and the anthropology of biomedicine to inquire into the material-discursive practices of global health, their consequences and contestations. But then again, as Labonté shows, one does not have to be an economist to see that hopes for a COVID-19-induced rupture of (economic) policy orthodoxy seem to have been premature. Not for the first time, the doomsday machine appears more like an unstoppable juggernaut.

 Insisting on the importance of economic inequities – in addition to colonialism, racism, sexism, classism, ageism, ableism, homophobia and transphobia – as stratifiers of health risks is thus an important intervention in itself. Indeed, in the early phase of the pandemic, the unprecedented and haphazard nature of hastily-implemented worldwide containment measures sparked lively commentary on the societal ‘fault lines’ exposed by COVID-19. But as the pandemic dragged on, public discourse seemed to move on. With the rollout of COVID-19 vaccines, media coverage and public attention also shifted onto the deficiencies of the COVAX initiative, inadvertently narrowing the issue of equity to a question of the unequal distribution of biomedical products. And yet, as my colleagues and I put it elsewhere, “inequities are not just the result of what happens when systems ‘fail.’ Rather, inequities are often the result of—and are refracted through—the way systems are set up and operate.”^6^ This means that ongoing efforts to boost vaccine manufacturing capacity in low- and middle-income countries are undoubtedly an important part of building a ‘new public health order,’ of the kind demanded by Africa Centres for Disease Control and Prevention Director John Nkengasong and colleagues.^7^ But so must be the insight that efforts to address health inequities need to go far beyond ensuring equitable access to healthcare technologies, however important this is.

 In the United Kingdom, Michael Marmot’s 2020 report *Build Back Fairer: The COVID-19 Marmot Review*^8^ has offered a devastating resumé of the structural inequalities that have driven the differential impact of the pandemic for different population groups: the United Kingdom experienced not just one of the highest level of ‘excess deaths’ in Europe, but data also showed stark economic and racial inequalities in mortality risk.^9^ As the report makes clear, such health inequities are driven by ‘causes of the causes of the causes,’ such as structural racism, as well as distinct policy failures: as the report argues, the United Kingdom entered the pandemic after 10 years of Conservative government that left “public services in a depleted state and its tax and benefit system regeared to the disadvantage of lower income groups.”^8^ For example, widespread cutbacks to government spending have been argued to not only have left the National Health Service ill-prepared to deal with the COVID-19 pandemic,^9^ but also led to rising child poverty, food insecurity and homelessness.^8^ Indeed, more than a decade after the publication of the *Final Report of the Commission on Social Determinants of Health*, the importance of paying attention to the structural drivers of ill health seems to have become widely accepted, at the same time that an appreciation of individual and population-level (health) inequities has failed to translate into a radical transformation of those political-economic systems that differently distribute power and resources.

 In their recently published treatise *Unprecedented? How COVID-19 revealed the politics of our economy*, Davies and colleagues note that “(o)nly during the periods of the deepest uncertainty do the true underpinnings of the system become visible.”^10^ They describe COVID-19 as a collision of the unexpected with the predictable: whereas the virus itself was novel and worldwide mitigation measures unprecedented, hastily implemented policies largely reiterated who and what matters in our current global, capitalist, economic system and revealed the “extraordinary social and political sacrifices and interventions that are made to sustain it.”^10^ One particularly revealing example, also picked up by Labonté, is how, after a decade of austerity imposed following the 2008 financial crash, the COVID-19 pandemic suddenly occasioned an outpouring of public funds to mitigate the crisis. In the United Kingdom, alongside other G7 economies, government borrowing rose to over 100% of gross domestic product, the highest level since 1963^11^ to fund, among other things, a huge economic rescue package to counterbalance the effects of government imposed lockdowns. But rather than celebrating a ‘roaring back of the state,’ it is important to highlight that not everyone benefitted equally from the huge injection of public monies, as lockdowns were only made possible because an underpaid and racialised workforce – health workers, shopkeepers, public transport staff, delivery drivers, etc – kept countries’ critical infrastructures going while bearing the greatest (health) risk as they became the frontline of countries’ pandemic response. At the same time, those who already owned assets saw their wealth multiply, not least as property prices and stock markets continued to soar.

 ‘Rentier capitalism’ is the term used by Brett Christophers and others to describe this system that rewards ownership of income-generating assets rather than, say, producing things.^12^ But although even proponents of the virtues of capitalism increasingly acknowledge the inequalities perpetuated by the growing disjuncture between capital- and production-based income,^13^ post-pandemic proclamations of ‘building back better’ have largely remained tethered to programs of tinkering around the edges rather than radical transformation. One example provided by Labonté is the resurrection of calls for a ‘stakeholder capitalism’ that sees companies shift focus from maximising shareholder value to creating long-term societal benefits. The World Economic Forum’s vision for stakeholder capitalism centres around the idea of multi-stakeholder platforms, which Labonté also recalls, has reignited long-standing concerns about the incursion of private sector actors and strategies into the global health governance sphere. Indeed, the COVAX scheme has arguably been the most prominent example of such a platform that, championed by Bill Gates as global health’s most renowned messenger of a benevolent capitalism, promised to leverage corporate power to tackle the health inequalities – and yet ultimately missed its own targets while being accused of eschewing public accountability.^14^

 One of the stakeholder capitalism’s blindspots, as a recent report argues, is that it disregards the ongoing trend towards corporate monopolisation and the associated accumulation of economic and political power that corporations are able to exercise.^15^ Indeed, what the COVID-19 pandemic has arguably underlined is that states do not harness corporate power (the tired justification for the privatisation of national assets and public services) but rather enable and defend it. In the health space, this became clear in the way wealthy country governments underwrote the financial risk of vaccine R&D and safeguarded the profit of pharmaceutical companies while propping up an international IP system that thwarts the transnational flow of life-saving products and know-how.^16^

 Even as it becomes increasingly obvious that COVID-19 has not, in fact, disrupted the status quo, one of the virtues of Labonté’s editorial is its refusal to capitulate and its insistence on an alternative future pursued through a number of tangible economic policy proposals. Among Labonté’s concrete suggestions are more progressive tax regimes (higher tax burdens for the better-off, stronger regulations to address tax evasion, a financial transaction tax), fiscal tools (‘modern monetary theory’), International Monetary Fund reforms, and a transition to a ‘de-growth’ economy. Among these, modern monetary theory – the proposition that there are, principally and under certain conditions, no fiscal limits to government spending – involves what is arguably the most innovative but also the most contentious set of recent economic ideas. But after the 2008 financial crash and post-COVID-19, it must be a legitimate question to ask why the public balance sheet can be used to bail out the economy by ensuring the liquidity of the market but not to improve working conditions, safeguard public services, re-common critical infrastructures, and fund the transition to a zero-carbon economy.

 Unfortunately, at least in the United Kingdom, we recently witnessed not the dismantling of the capitalist juggernaut but the Conservative government’s attempt at turbocharging it – with Liz Truss and her former chancellor proposing to cut funds for public services and taxes for high earners in the name of unleashing economic growth. And yet, the sustained backlash that these proposals caused may also be seen, optimistically, as a sign of a spreading discontent and an increasingly pandemic awareness of the inequalities and injustices at the heart of our dominant economic system. As Davies and colleagues note, “(t)hings that become visible, for while, can leave a residue.”^10^ Even if the COVID-19 might thus not have (yet) provided enough impetus for radical change, there is hope that its exposure of who and what gets to matter under capitalism will feed a collective desire for progressive social, economic and environmental justice-oriented politics.

 One of the most hopeful developments during the COVID-19 pandemic was the emergence of new forms of solidarity and care as well as a new wave of political protests. While I therefore agree with the sentiment of the call, at the heart of Labonté’s editorial, for the global public health community to support worldwide activist movements that, in turn, can put pressure on politicians and decision-makers, this could have perhaps been pushed further. As researchers, educators, colleagues, line-managers, supervisors, mentors and as citizens, we are not just part of the system but also already in a position to make a difference. Let’s make sure that a legacy of the pandemic is not just a legion of armchair epidemiologists – and, in my case, armchair economists – but a community of shoe-leather activists as well.

## Ethical issues

 Not applicable.

## Competing interests

 Author declares that she has no competing interests.

## Author’s contribution

 NJ is the single author of the paper.
